# Prolapsus of the Ovaries*Read before the Central New York Medical Association.

**Published:** 1880-02

**Authors:** Darwin Colvin

**Affiliations:** Clyde, N. Y.


### PROLAPSUS OF THE OVARIES.*

BY DARWIN COLVIN, M. D., OF CLYDE, N. Y.

THE following case came under my notice on the 8th of July last, and has been attended with much professional interest. Mrs. B., a widow, aged 29 years, while walking in the street, was suddenly seized with excruciating pain.

When I saw her she referred her suffering to the inguinal region and the anterior aspect of the thigh, in the track of the anterior crural nerve, and attended by a sense of fainting. She was pale, with a feeble and preternaturally slow pulse. In obtaining a history of her complaints, I learned that for two years past she had at times, more or less pain attending locomotion. At other times, when her bowels were unusually constipated,

* Read before the Central New York Medical Association.

she would have a throbbing sensation, sometimes amounting to severe pain during defecation. At another time, the pain would shoot out from the groin in various directions. Accompanying the symptoms was great depression of spirits, allied to hypochondria.

She had been married three years, yet never pregnant, the above symptoms supervening upon her married life. She suffered also from dysmenorrhœa, her menstrual periods lasting about a week. Upon examination I found the uterus much displaced downwards; to the left of the cervix my finger came in contact with a small tumor, exceedingly tender, and when pressed upon, exquisitely painful. In more fully examining the tumor, it immediately slipped from under the finger, when the patient shrieked. I could not distinctly outline the other ovary, it not having, as Prof. Goodell so intelligently describes it, yet been "pinched."

Although I had been treating her for symptoms simulating uterine neuralgia, yet her complaints were never of that character which seemed imperatively to demand a vaginal examination. Perhaps I came too readily to that conclusion; yet, when from necessity, symptoms referable to the sexual organs were spoken of, an excessive modesty and shyness on her part seemed to repel any earnest effort in that direction. Without a complete evacuation of the rectum once in twenty-four hours, she would, after from thirty to thirty-six hours, begin to complain of severe neuralgic pain in the groin and down the genito-crural nerve. Still, there had never been any complaint of pain in locomotion, nor sudden seizures of the same.

As to the causes of the displacements of these organs and the treatment, I can offer nothing original with myself, and, with one or two exceptions, I can find but little literature on the subject. Lawson Tait says, "the ovaries are liable to certain displacements which may give rise to many disagreeable symptoms without any actual disease of the glands. Thus one or both ovaries may, by a relaxation of their peritoneal investments, drop into the retro-uterine cul-de-sac, and there be a source of great trouble.

This will be especially the case, if, at the same time, there is retroflexion or retroversion of the uterus, for I have known such a displacement of an ovary utterly to prevent the application of an apparatus for the replacement of the uterus, and cause so much suffering as almost to make us discuss the question of ovariotomy.

In such displacements, pressure on the gland gives rise to the same sickness and faintness as pressure on the testicle produces in the male, and the passage of a hard motion will give rise sometimes to most alarming symptoms.

He says nothing further relative to the form of displacement under consideration.

It seems to have been left for Prof. Goodell, in a clinical lecture, to place the profession under lasting obligations so far as the etiology and treatment of this unique displacement is concerned.

He says, "any cause tending to a lasting congestion of the reproductive apparatus, is very likely to lead to a prolapse of the ovaries. A torn cervix, an arrest of involution after labor, any backward displacement of the womb, you may find it in barren women. The reason is this: In sterile women the lack of pregnancy and suckling prevents that much needed break in the constantly recurring catamenia, and the physiological congestions of the womb augmented by the sexual congestions, are, by ceaseless repetition, liable to become pathological."

Perverted sexual relations, and perverted sexual excitations, are by no means rare causes of this trouble.

For instance, I have repeatedly discovered the ovaries low down in women who were shirking maternity.

Here an over-stimulation of the whole reproductive apparatus is kept up both by the enforced sterility, and by some of the preventive measures employed, which awaken the sexual instinct without appeasing it. So repeated erectility from selfabuse, by ending in a passive congestion of the womb and of the ovaries, tends to these dislocations.

I have seen several cases of prolapse of the ovaries from this cause.

It is unnecessary to further enlarge upon the causes of this difficulty.

Undoubtedly the usual symptoms of this displacement are, first: Pain in locomotion, as the ovary now lies between the womb and the sacrum, it gets pinched between them at every step.

Second: A throbbing pain while the rectum is loaded, increased during the act of defecation.

Third: Paroxysms of pain shooting out from the groin.

Fourth: Mental despondency.

Prof. Goodell gives another symptom attending the dislocation, to wit: painful coition.

The indications for treatment would seem to be whatever will have a tendency to lessen the engorgement of the reproductive organs.

The treatment recommended by him is brom. potassium grs. xxx. tinct. digitalis gtt. x. three times a day in a tablespoonful of the comp. infusion of gentian; after two weeks, alteratives as the corros. chlo. of mercury, are usually necessary.

One of the most, if not the most important agent for the purpose of keeping up the ovaries, and one which I made use of in this case, is the knee-breast posture devised by Dr. Campbell of Georgia. As Prof. Goodell recommends it to be more thoroughly carried out than I did (not having then seen his paper), I will give his instructions verbatim:

"Two or three times a day, or more frequently, if needful, the patient should unhook her dress, loosen her underclothing and kneel on her bed. Her body is then bent forward until the breast is brought down to the surface of the bed, while her head is turned to one side and supported in the palm of her left hand. Her knees should be about ten inches apart, and the thighs perpendicular to the bed.

"If she now refrains from straining, and breathes naturally, a reversal of gravity will be established.

"With the fingers of her free hand, she will next open her vulva. Air will rush in, and the abdomen and its contents will at once sag down. This will, of course, draw up the womb and the displaced ovaries out of the pelvic canal. As it is rather awkward for a woman, while in this posture, to free one hand and reach the vulva, Dr. Campbell advises that, previously to taking this attitude, she should insert into the vagina a small glass tube, open at both ends, and long enough to project externally. This will leave an air-way and dispense with the use of the fingers. A good substitute will be found in the empty barrel of the old-fashioned cylindrical "female syringe," as it is called.

"After staying in this posture for a few minutes, the woman will remove the tube, and slowly turn over on her side, where she will lie as long as she can. Such constant replacements are of great service, for they lessen the throbbing; they give the limp ligaments a chance of shrinking, and they teach the ovaries good habits of staying at home."

The course pursued with my patient was, with perhaps the exception of the knee-breast position, one which would readily suggest itself to any practitioner, varying the specific agents as circumstances might seem to require. I only directed the knee-breast posture to be observed once daily, and that upon retiring, with directions that she occupy, at *all times*, a bed, the foot of which should be elevated eight inches; also, that she observe the recumbent position as much as was convenient. I am now well convinced that had I required the observance of the knee-breast position twice or thrice daily, my case would have improved more rapidly, as her condition was approaching anæmia

As it was, her general health was materially tonified, and the position of the misplaced ovary was very much improved on the 20th of August, when she was about to make a necessary though temporary change of residence.

